# Clinical and Etiological Spectrum of Hypokalemic Periodic Paralysis in a Tertiary Care Hospital in Pakistan

**DOI:** 10.7759/cureus.3921

**Published:** 2019-01-19

**Authors:** Zumar Sardar, Kh. Ahmad Furqan Waheed, M. Athar Javed, Faisal Akhtar, Syed Rizwan A Bokhari

**Affiliations:** 1 Neurology, King Edward Medical University / Mayo Hospital, Lahore, PAK; 2 Neurology, Ochsner Health System, New Orleans, USA; 3 Nephrology, Tulane University School of Medicine, New Orleans, USA

**Keywords:** hypokalemic periodic paralysis, hypokalemic paralysis, low serum potassium

## Abstract

Introduction

Hypokalemic periodic paralysis (HPP) is characterized by muscle weakness secondary to low serum potassium levels. It may be primary in origin or there may be secondary causes like thyrotoxic periodic paralysis, renal or suprarenal causes, or non-renal causes like gastroenteritis.

Aim

To study the etiology, clinical manifestations, and outcome after therapy of patients with hypokalemic paralysis.

Methodology

The study was conducted from January 2016 to December 2016. Patients fulfilling the diagnostic criteria for hypokalemic paralysis, i.e., flaccid muscle weakness involving two or more limb muscles due to serum potassium < 3.5 mmol/L and with no objective sensory signs were included in the study. Relevant investigations were done. Those with other causes of flaccid weakness or on diuretic therapy were excluded from the study. Data was analyzed using SPSS Version 20.0 (IBM Corp., Armonk, NY).

Results

In our study, 14 patients out of a total of 18 (14/18, i.e., 77.78%) were male and 4/18 (22.22%) were female [Male: Female ratio: 3.5:1]. The mean age of onset of HPP in males (29.5±10.14 yrs.) was lesser than that of females (41±10.8 yrs.), but this difference was statistically not significant (p<0.066). In the entire sample there were 15/18 cases (83.33%) of primary and 3/18 (16.67%) cases of secondary HPP [2/3 had thyrotoxic periodic paralysis and 1/3 had gastroenteritis]. Furthermore, 12/18 patients (66.66%) had symmetrical weakness (five cases of paraparesis and all were male; seven cases of quadriparesis: six males and one female) and 6/18 (33.33%) had asymmetrical weakness (two paraparesis: one male, one female; four quadriparesis: two males, two females). Statistically, no significant difference (p<0.709) was seen in those with symmetrical versus those with asymmetrical weakness. In this study 7/18 (38.89%) cases had absent, 1/18 (5.55%) had diminished, and 10/18 (55.55%) cases had intact deep tendon reflexes. None of the cases had cranial, bulbar, or respiratory involvement. The mean serum potassium of sample was 3.18±0.5 standard deviation (SD). The reduction in serum potassium was moderate (2.5-3.5 mmol/L) in primary and severe (<2.5 mmol/L) in secondary HPP. Those with quadriparesis had severe hypokalemia with a mean serum potassium of 2.1 mmol/L. Only 3/18 patients had concomitant magnesium deficiency. Patients given intravenous potassium replacement (except one with moderate hypokalemia and given oral replacement) recovered dramatically. The mean recovery time was 38.6±20.3 hours. The recovery time in quadriparesis was about 24 hours and in paraparesis was 12 hours. Only one patient with thyrotoxic periodic paralysis (TPP) and with severe serum potassium deficiency (0.9 meq/L) died due to cardiac arrhythmia. No atypical presentation was seen.

Conclusion

HPP has male preponderance. The age of onset of HPP is earlier in males than in females. Moreover, males are more prone to have symmetrical weakness. Asymmetrical weakness has almost an equal gender distribution. Primary hypokalemic paralysis is more frequent than secondary. Thyrotoxic periodic paralysis is the commonest cause of secondary periodic paralysis. The recovery time in quadriparesis is almost double the recovery time in paraparesis. Respiratory involvement is rare. HPP is an important differential in the diagnosis of acute flaccid muscle weakness. It should be promptly addressed to prevent recurrence of paralysis.

## Introduction

Hypokalemic paralysis is one of the common causes of acute flaccid paralysis that is characterized by muscle weakness due to low serum potassium levels [1]. Hypokalemic paralysis can be primary or secondary. Hypokalemic periodic paralysis (HPP), a calcium channelopathy, may be familial with autosomal dominant inheritance or sporadic [1]. Secondary causes of hypokalemic paralysis include renal causes (renal tubular acidosis, Gitelman syndrome, and primary hyperaldosteronism), endocrine causes (hyperthyroid periodic paralysis), and hypokalemia secondary to gastrointestinal losses (diarrhea) [2].

Familial hypokalemic paralysis is one of the most important causes of hypokalemic periodic paralysis among Caucasians [3], and thyrotoxic periodic paralysis is the leading cause of hypokalemic paralysis in the Asian population with male to female ratio of approximately 70:1. In Asian males, hypokalemic periodic paralysis (PP) affects 2-10% of thyrotoxic patients [4].

The age of onset of hypokalemic periodic paralysis is mostly in the first to second decade [3]. Hypokalemic periodic paralysis is a genetic disorder caused by mutation in voltage gated calcium channel CACNA1S gene on chromosome 1q [1,3,4]. Over the past decade, mutations in genes encoding three ion channels CACN1S, SCN4, and KCNJ2 have been identified and accounted for at least 70% of cases of periodic paralysis [1,2,4]. Hypokalemic periodic paralysis is characterized by recurrent attacks of skeletal muscle weakness lasting minutes to hours with associated hypokalemia [2]. HPP usually spares bulbar, ocular, and respiratory muscles. Hypokalemia is precipitated by stress, carbohydrate-rich meal, infection, glucose infusion, hypothermia, anesthesia, strenuous exercise, metabolic alkalosis, and steroids [5].

Thyrotoxic periodic paralysis is related to loss of function mutation of the skeletal muscle-specific inward rectifying K channel (Kir). Kir2.6 is associated with decreased outward K efflux in skeletal muscle from either channeled mutation or hormone (insulin, adrenaline), leading to a vicious cycle of hypokalemia, which in turn leads to sodium (Na) inactivation, skeletal muscle weakness, or paralysis. By successful treatment of thyrotoxicosis, symptoms of hypokalemic paralysis disappear [6].

Renal causes of hypokalemic paralysis are well known. Renal tubular acidosis (RTA) is a recognized cause of severe hypokalemia [5]. RTA and severe hypokalemia are associated with medullary sponge kidney, cystic kidney disease, and nephrocalcinosis [7]. Gastrointestinal potassium losses that occur due to heavy fluid losses through the biliary tract or bowel are not uncommon [8].

There is only one case report of a patient with hypokalemic paralysis caused by falciparum malaria, who recovered with parenteral quinine, published in the Pakistan Armed Forces Medical Journal [9]. According to previous case studies in literature, some patients may go on to develop permanent proximal muscle weakness after years with periodic paralysis [5].

The mainstay of treatment in hypokalemic periodic paralysis is potassium replacement and acetazolamide, a carbonic anhydrase inhibitor. Hypokalemic paralysis is usually overlooked in patients with acute flaccid paralysis in the emergency room [10].

## Materials and methods

The present study is a retrospective chart review done over a period of one year from January 2016 to December 2016. All patients admitted in the neurology ward in King Edward Medical University with acute flaccid paralysis with hypokalemia (serum potassium <3.5) involving two or more limbs without sphincter and sensory disturbances were included in the study. Patients with other causes of acute flaccid weakness, e.g. Guillain-Barré Syndrome (GBS), myasthenia crisis, and on diuretic therapy were precluded from the study. Medical history, previous episodes of similar weakness, hyperthyroidism, diarrhea, vomiting, renal disease, and drug intake were noted. Any history of similar illness in the family and precipitating factors were enquired about and noted. Complete neurological examination scores including muscle tone, assessment of power using Medical Research Council (MRC) scale, and deep tendon reflexes were noted. Any atypical presentation was also noted, e.g. facial, bulbar, or respiratory muscle involvement. Labs including complete blood count (CBC), serum potassium, serum sodium, serum calcium, serum bicarbonate, thyroid profile (T3, T4, thyroid-stimulating hormone (TSH)), and electrocardiogram (ECG) were obtained on the day of admission.

All patients were treated with potassium supplements—oral (500 mg at eight-hour intervals) or intravenous (IV) (10 meq/hour) depending upon their serum potassium levels and severity of clinical manifestations (extreme weakness). Patients with idiopathic periodic paralysis were started on acetazolamide (250 mg tid) and the dose was titrated according to the response.

Patients were divided into groups: the first group was patients with primary idiopathic hypokalemic periodic paralysis and the second group was patients with secondary hypokalemic periodic paralysis (thyrotoxic periodic paralysis, renal tubular acidosis, and gastroenteritis).

Statistical analysis

Continuous variables were expressed as mean ± standard deviation. A p-value < 0.05 was considered statistically significant and a p-value < 0.01 was considered statistically highly significant. All statistical analysis was conducted using SPSS Version 20.0 (IBM Corp., Armonk, NY).

## Results

We enrolled 18 patients (mean age 35±15) and 14 patients (77.78%) were males, [male: female ratio: 3.5:1]. The mean age of onset of HPP in males was (29.5±10.14 yrs.) as compared to females (41±10.8 yrs); however, this difference was statistically not significant (p<0.066). Off these patients, 15 (83.33%) patients had primary HPP, while three (16.67%) cases had secondary HPP [2/3 with thyrotoxic periodic paralysis and 1/3 cases was secondary to gastroenteritis].

Out of total 18 patients, symmetrical weakness was found in 12 (66.66%) patients with predominance in male patients (five cases of paraparesis and all were male; seven cases of quadriparesis: six males and one female). Six (33.33%) patients had asymmetrical weakness (two paraparesis: one male, one female; four quadriparesis: two males, two females). Statistically, no significant difference (p<0.709) was seen in those with symmetrical vs. asymmetrical weakness. Deep tendon reflexes were absent in seven (38.89%) patients, diminished in one patient (5.55%), and intact in the remaining 10 (55.55%). None of the cases had cranial, bulbar, or respiratory involvement. The mean serum potassium of sample was 3.18±0.5 SD.

The reduction in serum potassium was moderate (2.5-3.5 mmol/L) in primary and severe (<2.5 mmol/L) in secondary HPP. Those with quadriparesis had severe hypokalemia with a mean serum potassium of 2.1 mmol/L. Concomitant magnesium deficiency was observed in 3/18 (17%) patients. These patients were treated with intravenous potassium replacement with dramatic recovery. The mean recovery time was 38.6±20.3 hours. Recovery time in patients with quadriparesis was about 24 hours and in those with paraparesis was around 12 hours. Only one patient with thyrotoxic periodic paralysis (TPP) and with severe serum potassium deficiency (0.9 meq/L) died due to cardiac arrhythmia. No atypical presentation was seen in the present study.

## Discussion

The hypokalemic paralysis of 15 patients in our study was primary idiopathic hypokalemic periodic paralysis. Secondary hypokalemic paralysis occurred in three (16.67%) patients, thyrotoxic periodic paralysis (11.5%), and diarrhea (5.6%). The etiology of hypokalemic paralysis is varied across different ethnicities and geographical areas [1,3]. In a study from North Korea, most of the cases of hypokalemic paralysis were due to secondary hypokalemic paralysis, and among secondary causes, thyrotoxic periodic paralysis was found more frequently than others [5].

There was a significant difference in serum potassium levels in the two groups; patients with secondary hypokalemic paralysis had more severe hypokalemia than primary hypokalemic periodic paralysis.

Despite the higher incidence of thyrotoxic periodic paralysis in males [11], in our study there was one male and one female with TPP. Male preponderance of thyroid periodic paralysis is hypothesized to be due to testosterone levels in blood [4,6]. TPP is curable once acute thyrotoxicosis is resolved.

Overall, the mean recovery time was 38.6±20.3 hours. Recovery time in patients with quadriparesis was about 24 hours and those with paraparesis was around 12 hours.

Rarely does the thyrotoxic or hypokalemic periodic paralysis affect bulbar, ocular muscles, or cranial nerves [2,3,7,11]. In previous studies, the need for ventilator support in hypokalemic and thyrotoxic periodic paralysis has been reported [11]. None of our patients had any involvement of eye muscles or cranial nerves, neither did they need ventilator support.

Other electrolyte abnormalities in thyrotoxic periodic paralysis have been reported, e.g. transient hypomagnesemia and hypophosphatemia, which tend to resolve once acute thyrotoxicosis settles [4,7]. In our study, one female patient with TPP had concomitant magnesium deficiency that was documented to be 0.9 meq/L and that patient died of cardiac arrhythmia.

There were increased occurrences of recurrent attacks in hypokalemic periodic paralysis than in thyrotoxic periodic paralysis.

Treatment depends upon the severity of attack. Minor attacks with mild hypokalemia tend to resolve spontaneously. Management of hypokalemic periodic paralysis includes accurate diagnosis, optimum potassium replacement, choice of diuretic prophylaxis, identification of triggering factors, and managing the issues in pregnancy [12]. Potassium chloride is the preferred salt given at 0.5-1 mEq/kg for acute attacks [13]. In the hypokalemic periodic paralysis group, 43% (7/16) had paraparesis, and 56% (9/16) had quadriparesis. Typical hypokalemic periodic paralysis causes paraparesis. The sensory system, bowel, and bladder remain intact [10]. Acetazolamide or potassium sparing diuretics decrease the frequency of attacks and severity of attacks in future but are of no use during acute attack hypokalemic paralysis [10]. Oral potassium in the form of solution, powder dissolved in water, or sustained release tablets have a role in its management [13]. In cases where patients suffer from recurring morning attacks of paralysis, a trial of sustained release tablet taken at the time of sleep is warranted [10]. In this study all patients responded well to intravenous potassium. Patients with moderate to severe hypokalemia require intravenous potassium [14]. There was only one patient in the primary group in whom there was a spontaneous recovery. Potassium can be given orally for prophylaxis as well before strenuous exercise or carbohydrate load [10]. During an acute attack, quicker results can be obtained by giving an oral solution of potassium;  response is seen within 30 minutes after administration [14], and total dose of potassium given should not exceed 240 meq/l in 24 hours. Glucose or dextrose should not be used as the solvent for oral solution, instead mannitol is considered a preferred solvent [13]. Chronic administration of potassium supplements can lead to gastric irritation from locally high salt concentration. This can be counteracted with concomitant proton pump inhibitors. Acetazolamide is given as maintenance therapy. Carbonic anhydrase inhibitors are potassium wasting; occasional potassium supplements, even in the absence of attack, is warranted. If carbonic anhydrase inhibitors are not available, then spironolactone is an option [15].

Management of thyrotoxic periodic paralysis depends on the adequate management of hyperthyroid state and correction of electrolyte abnormalities associated with it [16]. All three patients in the secondary group were also given intravenous potassium, and there was one mortality due to cardiac arrhythmia in the TPP group. Intravenous (IV) replacement of potassium is done in TPP patients to prevent cardiopulmonary disturbances. However, with potassium replacement there is a danger of rebound hyperkalemia, usually after recovery of muscle weakness. Beta adrenergic blockers, e.g. propranolol, are given to prevent rebound hyperkalemia and hasten recovery [16].

Myopathy develops in at least 25% of the affected individuals and may lead to permanent muscle weakness later in life [10]. No fixed, progressive proximal myopathy was found in our patients.

Limitations of the study

The sample size of the study was small to comment on the most frequent group as a cause of hypokalemic paralysis in our population. Transtubular potassium concentrating gradient, potassium creatinine ratio, urinary potassium, and transtubular potassium concentrating gradient during paralytic attack were not done due to the cost and non-availability of these tests at our center.

## Conclusions

Hypokalemic paralysis should always be kept in mind when making a differential diagnosis for acute flaccid paralysis. In all patients, serum electrolytes panel, ECG, and thyroid profile should be done at admission in the intensive care unit (ICU). Immediate treatment should be started either orally or intravenously on the diagnosis of hypokalemic paralysis. Monitoring for cardiac arrhythmias should be done vigilantly.
